# Role of altered esophageal intraluminal baseline impedance levels in patients with gatroesophageal reflux disease refractory to proton pump inhibitors

**DOI:** 10.1097/MD.0000000000004351

**Published:** 2016-08-19

**Authors:** Liuqin Jiang, Bixing Ye, Lin Lin, Ying Wang, Meifeng Wang

**Affiliations:** a Department of Gastroenterology, The First Affiliated Hospital of Nanjing Medical University; b Department of Gastroenterology, Jiangsu Province Official Hospital, Nanjing, Jiangsu, China.

**Keywords:** acid reflux, baseline impedance level, MII-pH monitoring, nonacid reflux, refractory gastroesophageal reflux disease

## Abstract

Numerous studies have investigated utility of esophageal intraluminal baseline impedance levels (BILs) in gastroesophageal reflux disease (GERD). However, effect of BILs in refractory GERD (RGERD) has not been well investigated. The aim of this study is to evaluate role of BILs in RGERD patients. Total 62 subjects with refractory gastroesophageal reflux symptoms underwent 24-hour impedance-pH monitoring and gastroendoscopy. Distal BILs in acid reflux type were significantly lower than those in nonacid reflux type and functional heartburn (FH) group. Distal BILs of reflux esophagitis (RE) patients were lower than those of nonerosive reflux disease (NERD) patients, while there were no statistical significance between 2 groups. Patients with severe esophagitis had lower distal BILs than those with mild esophagitis and NERD patients, and patients with severe esophagitis in acid reflux type had the lowest distal BILs. Distal BILs were significantly negatively correlated with DeMeester score, episodes of acid reflux, and acid exposure time, but no correlated with episodes of nonacid reflux. Characteristics of BILs in RGERD patients were similar with those in GERD patients, but might be more complicated. Evaluating BILs in RGERD patients could achieve a better understanding of pathophysiology in RGERD.

## 1 Introduction

Gastroesophageal reflux disease (GERD) is considered as refractory when the improvement of symptoms with a standard proton pump inhibitors (PPIs) treatment for 12 weeks is <50%.^[1]^ The pathological mechanism of refractory gastroesophageal reflux disease (RGERD) is multifactorial and remaining unclear. Multiple elements have been proposed for play in the genesis of PPI-refractory symptoms: insufficient suppression of acid reflux; association with nonacid reflux; abnormal esophageal motility; esophageal hypersensitivity; and psychological factors.^[2,3]^

Twenty four hour multichannel intraluminal impedance and pH monitoring (MII-pH monitoring) plays a very important role in detection of reflux episodes in GERD patients,^[4–6]^ and enables classification of RGERD into reflux-related disease, including acid reflux and nonacid reflux types, and including functional heartburn (FH).^[1,7]^ Although when there are no swallows or episodes of reflux, the resulting intraluminal baseline impedance level (BIL) reflects the primary electrical conductivity of the esophagus based on that the esophageal wall contacts directly with the MII-pH sensor catheter.

Previous studies, which focused on GERD and FH diseases, have revealed that BILs in GERD patients were lower than FH patients and healthy people, and demonstrated that BILs are correlated with acid exposure time (AET) and mucosal injury of the esophagus, and suggested that BILs are considered as an indicator of the esophageal mucosal integrity.^[8–12]^ However, role of BILs in the pathogenesis of RGERD has not been adequately investigated until now. Recent study in Japan demonstrated that among patients with PPI-refractory nonerosive reflux disease (NERD), acid reflux group had lower BILs compared with nonacid reflux group and FH group.^[13]^ Martinucci et al^[14]^ showed that BILs of FH patients negative response to PPIs were lower than those of responders.

We hypothesized that BILs are altered in certain segments of the esophagus in RGERD patients such as GERD patients, and that the alterations are associated with reflux events and esophageal function. The aims of this study were: to evaluate the composition of BILs from proximal to distal esophagus in RGERD patients and compare the BILs among patients with different reflux type; to evaluate their relation to different reflux events and acid-related parameters in RGERD patients; and to assess whether BILs are associated to erosive esophagitis.

## 2 Materials and methods

### 2.1 Study subjects

All patients referred to the First Affiliated Hospital of Nanjing Medical University outpatient clinics from January 2014 to December 2015 in this retrospective study. Inclusion criteria included age of 18 years or older and any gender. All patients had typical heartburn or regurgitation according to the Montreal consensus,^[15]^ lasting >6 months, and their reflux disease questionnaire scores were not less than 12.^[16]^ They met the criteria of RGERD which improvement of symptoms was <50% after they were treated with omeprazole 20 mg or rabeprazole 10 mg bid for at least 12 weeks.^[1]^ All subjects underwent endoscopy in 1 month before analysis, the degree of esophageal mucosal injury was graded according to Los Angeles classification (LA A–D).^[17]^ Patients were excluded if they had a history of peptic ulcer, gastrointestinal tumor or surgery, or severe esophageal motility disorders. In addition, subjects were also excluded if they were found to have abnormalities other than erosive esophagitis or chronic superficial gastritis by gastroendoscopy. The study was approved by the Ethical Committee of First Affiliated Hospital of Nanjing Medical University.

### 2.2 Esophageal multichannel intraluminal impedance and pH monitoring

Subjects of PPIs, H_2_-antagonist, or prokinetic drugs therapy which could influence esophageal motor function or gastric acid secretion at least 1 week underwent MII-pH monitoring using an ambulatory monitoring system (Given Imaging, Duluth, GA). The catheter was inserted into distal esophagus through nasal cavity. The catheter contained 6 impedance segments and 1 pH electrode sensor. The pH sensor was placed at 5 cm above the lower esophageal sphincter (LES) (which was located by manometry or traditional method), and 6 impedance values (z1, z2, z3, z4, z5, and z6) were recorded at 6 sites (17, 15, 9, 7, 5, and 3 cm above the LES, respectively).

### 2.3 Analysis for reflux parameters and BILs

The data of reflux events and parameters were measured by automatic analysis software of monitoring system. Two investigators (LJ and BY) blinded for the diagnosis of patients reviewed the tracings manually to ensure accurate detection and classification of reflux episodes and BILs. The meal periods were excluded from the analysis.

The following reflux parameters were analyzed in our study: the number and type of reflux episodes^[18]^; DeMeester score; and AET. Reflux type is usually divided into 3 categories: acid, weakly acid, and alkaline reflux. Therefore, in our study we characterized the reflux episodes as acid or nonacid (including weakly acid and alkaline), respectively, if the nadir pH reached <4.0 or was constantly ≥4.0. AET was defined as the distal esophageal total time with pH below 4, divided by the total time of monitoring. BILs were assessed in a manner blinded to the diagnostic results at 3 time points (around 00:00 am, 10:00 am, and 4:00 pm) between meals, avoiding close to any period of swallowing or reflux. Each single BIL represents the average level of 3 suitable baseline levels around each time point. We chose BILs from the 5th impedance site (z5, 5 cm above the LES) to analyze their correction with reflux parameters, where there was a pH sensor to ensure that BILs were selected just when pH > 6.

### 2.4 Definition of acid reflux type and nonacid reflux type on the basis of MII-pH monitoring

In our study, the patients would be classified as having RGERD if reflux parameters were positive according to pH monitoring. On the basis of endoscopy results, RGERD patients were divided into reflux esophagitis (RE) and NERD. We subclassified RGERD patients into acid reflux and nonacid reflux types according to the level of AET and acid reflux episodes. Patients with RGERD were considered as acid reflux type if there were abnormal AET (>4.2%) and acid reflux episodes. Other RGERD patients were considered as nonacid reflux type. Patients with typical heartburn, negative endoscopy, and negative pH monitoring are classified as FH.^[6,19,20]^

### 2.5 Statistical analysis

Data are expressed as means ± SD. We used Student *t* test when there were 2 groups being compared and analysis of variance for difference in mean values. Post hoc comparisons were performed using the LSD correction in the case of significant analysis of variance (ANOVA) results. Correlation between BILs from z5 and reflux parameters were performed with Spearman's rank test 2-tailed). A *P* < 0.05 was considered statistically significant. Statistical analysis was performed using SPSS version 19.0 for windows (SPSS, Chicago, IL).

## 3 Results

### 3.1 Demographic and clinical characteristics

Total 62 patients with refractory gastroesophageal reflux symptoms were enrolled (33 males, mean age 51.2 ± 14.4 years). All patients tolerated MII-pH monitoring and had no adverse events. Before MII-pH monitoring, 48 patients completed esophageal manometry to locate LES. RGERD was identified in 52 patients and was classified as acid reflux type (24 patients) and nonacid reflux type (28 patients). A total of 10(16.1%) patients were diagnosed as FH, 27 patients were diagnosed as RE (7 LA C/D and 20 LA A/B), and 25 patients were diagnosed as NERD. Detailed demographic data and MII-pH monitoring parameters are summarized in Table 1.

**Table 1:**
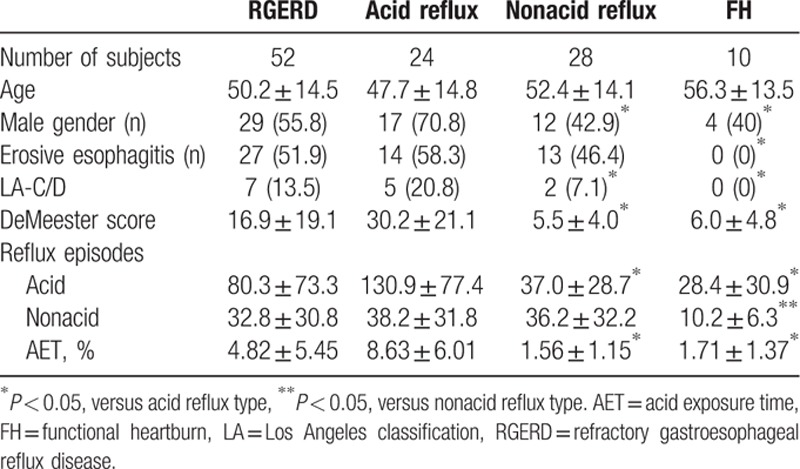
Baseline characteristics of the study groups.

### 3.2 BILs in different reflux type

There was a decreasing trend in mean BILs from proximal to distal esophagus in total RGERD patients, while only BILs from z6 were significantly lower than others from z1 to z5 (all *P* < 0.01). Acid reflux type's BILs decreased from z1 to z6, BILs from z5 were lower than those from z1 to z4 (all *P* < 0.01), and BILs from z6 were lower those from z1 to z5 (all *P* < 0.01), while there were no difference among BILs of each site in nonacid reflux type (all *P* > 0.05) (Table 2).

**Table 2:**
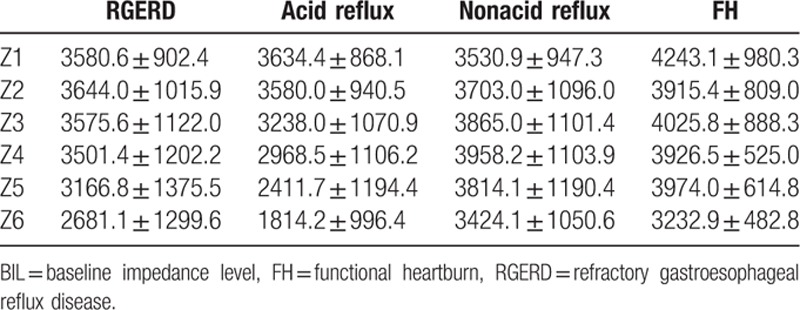
BILs (Ω) of all patients across groups from different site.

**Figure 1:**
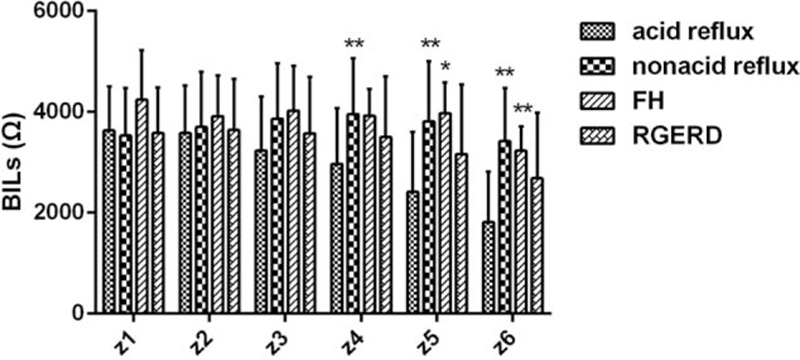
Baseline impedance levels (BILs) of each group from different site. Data were expressed as means (95% confidence intervals) versus acid reflux type ^∗^*P* < 0.05, ^∗∗^*P* < 0.01.

Figure 1 displayed the comparison of BILs across groups. There was no significant difference in each site's mean BILs between RGERD and FH patients. Also there was no significant difference in all sites’ BILs between nonacid reflux and FH group. BILs from z4 to z6 in acid reflux type were significantly lower than that in nonacid reflux type (all *P* < 0.01). The lower BILs from z5 to z6 compared with FH group were consistent in acid reflux type (*P* = 0.013, *P* = 0.009, respectively). BILs from z6 in acid reflux type were the lowest value among all groups.

### 3.3 BILs in RE and NERD group

BILs were lower in RE group than those in NERD group, but there was no statistical significance between 2 groups. DeMeester score, AET, and acid reflux episodes were significantly higher in LA C/D subgroup than those in LA A/B subgroup and NERD group. BILs from z4 to z6 were significantly lower in LA C/D subgroup than those in LA A/B subgroup and NERD group (all *P* < 0.05). Detailed characteristics of each group were summarized in Table 3.

**Table 3:**
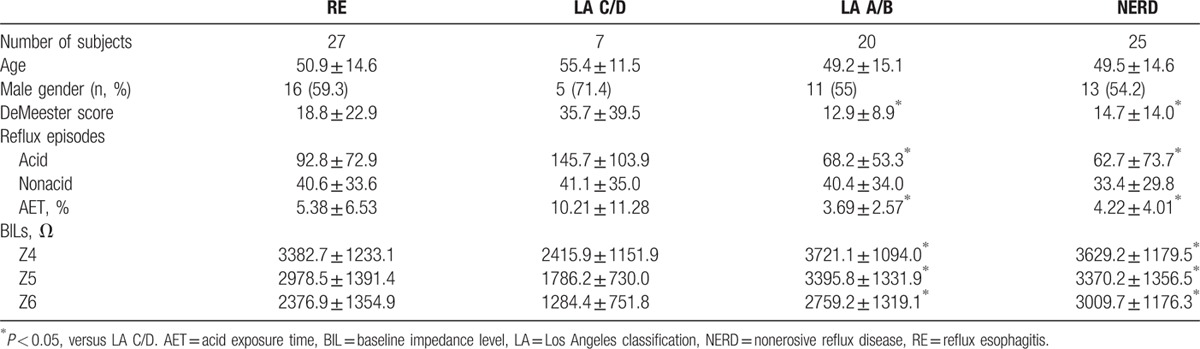
RE and NERD group's characteristics.

**Figure 2:**
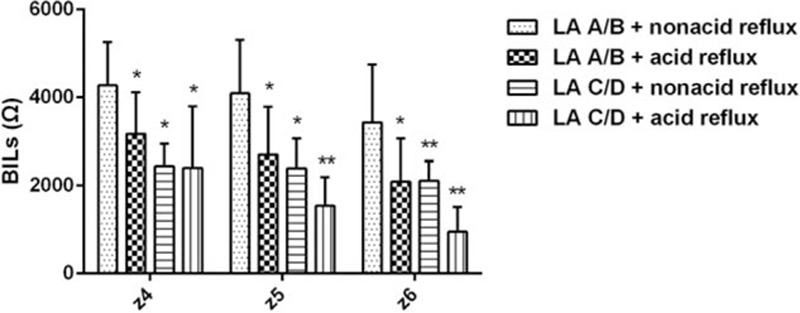
BILs from z4 to z6 with different degree esophagitis in acid reflux and nonacid reflux types. BILs from z4 to z6 in LA A/B + acid reflux, LA C/D + nonacid reflux, and LA C/D + acid reflux subgroups were lower significantly than those in LA A/B + nonacid reflux subgroup, and BILs from z6 in LA C/D + acid reflux subgroup were lowest versus LA A/B + nonacid reflux subgroup ^∗^*P* < 0.05, ^∗∗^*P* < 0.01. BIL = baseline impedance level, LA = Los Angeles classification.

Within acid reflux type, there were 5 LA C/D RE patients (LA C/D + acid reflux) and 10 LA A/B patients (LA A/B + acid reflux); within nonacid reflux type, there were 2 LA C/D patients (LA C/D + nonacid reflux) and 10 LA A/B patients (LA A/B + nonacid reflux). BILs with different degree esophagitis in different reflux type were summarized in Table 4. BILs from z4 to z6 in LA A/B + acid reflux, LA C/D + nonacid reflux, and LA C/D + acid reflux subgroups were lower significantly than those in LA A/B + nonacid reflux subgroup, and BILs from z6 in LA C/D + acid reflux subgroup were lowest among all subgroups (Fig. 2).

**Table 4:**
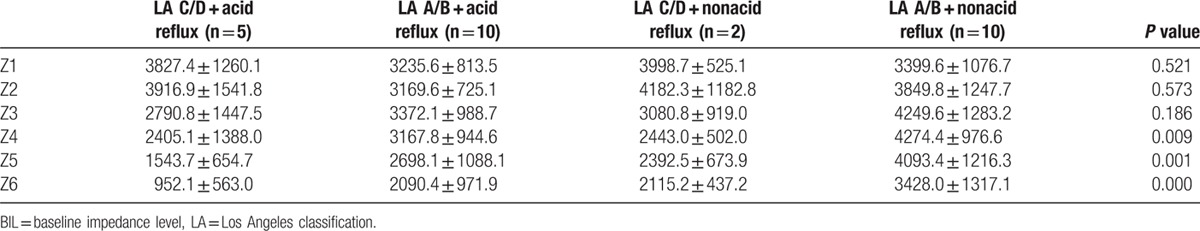
BILs with different degree esophageal inflammation in different reflux type.

### 3.4 Correlation between BILs from z5 and reflux-related parameters

**Figure 3:**
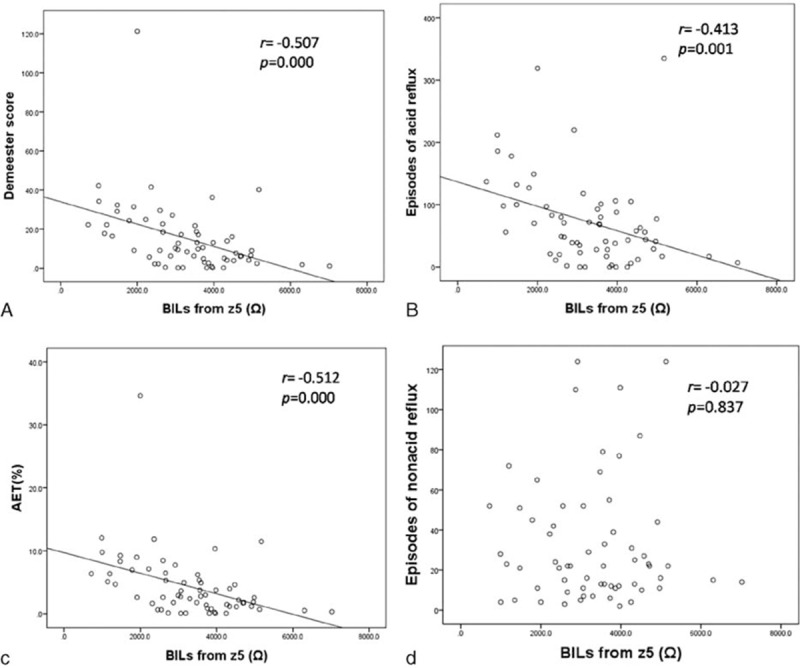
Correlation between BILs from z5 and reflux related parameters. (A) Correlation of DeMeester score and BILs from z5 (*r* = −0.507, *P* = 0.000, n = 62). (B) Correlation of episodes of acid reflux and BILs from z5 (*r* = −0.413, *P* = 0.001, n = 62). (C) Correlation of AET and BILs from z5 (*r* = −0.512, *P* = 0.000, n = 62). (D) Correlation of episodes of nonacid reflux and BILs from z5 (*r* = −0.027, *P* = 0.837, n = 62). AET = acid exposure time, BIL = baseline impedance level.

BILs from z5 were significantly negatively correlated with DeMeester score (*r* = −0.507, *P* = 0.000, n = 62) (Fig. 3A), with episodes of acid reflux (*r* = −0.413, *P* = 0.001, n = 62) (Fig. 3B), and with AET (*r* = −0.512, *P* = 0.000, n = 62) (Fig. 3C). Although BILs from z5 had no correlation with episodes of nonacid reflux (*r* = −0.027, *P* = 0.837, n = 62) (Fig. 3D).

## 4 Discussion

We acknowledged the possibility that some patients with refractory gastroesophageal reflux symptoms may have been misclassified owing to clinical examination limitations. Previous studies reported that patients with refractory gastroesophageal reflux symptoms often did not have GERD,^[1,3,21]^ and that those patients diagnosed as GERD were more related with nonacid reflux (weakly acid and alkali reflux).^[22–27]^ Consistent with the above studies, our findings showed 45.2% patients with refractory gastroesophageal reflux symptoms were associated with nonacid reflux and 16.1% patients were considered as FH. Our study specially aimed to determine role of BILs in RGERD patients.

Previous investigations demonstrated that BILs by using MII-pH monitoring in healthy subjects were in the range of thousands of Ohms,^[6,9,28]^ while in distal esophagus of patients with acid reflux or esophagitis were in the range of several hundreds of Ohms, and that distal esophageal BILs were significantly lower than proximal esophageal BILs.^[10,29]^ In our study, we found that there was a decreasing tendency in BILs from proximal esophagus to distal esophagus in RGERD patients and patients with acid reflux type. But the lowest distal BILs of RGERD patients were nearly 2 thousands of Ohms, which were higher than those from above-mentioned studies. We have yet to figure out a clear explanation for our findings above. Anyway, we believe that composition of BILs in RGERD patients was more complex than that in regular GERD patients, because long-term PPIs or other medicines usage could lead to mucosal inflammatory improvement or recovery and esophageal mucosal injury may be just one of pathogenic factors of RGERD but not the most important one. Furthermore, the design of this retrospective study and small sample size might be related with this result.

Several studies showed that distal BILs of GERD patients with pathological acid reflux were markedly lower than those of healthy volunteers.^[8,9]^ Zhong et al^[10]^ revealed that distal BILs in GERD patients with acid reflux were lowest, and followed by those with weakly acid reflux, alkali reflux, and normal population. Kandulski et al^[30]^ found that distal BILs in GERD patients were lower than those in FH patients. In our study, there were no difference in BILs between nonacid reflux group and FH group. We found total episodes of abnormal reflux in nonacid reflux group were significantly less than those in acid reflux group and similar with FH group. We think that less episodes of abnormal reflux could lead to relatively mild injury, and that a lot of medicines usage in RGERD patients could improve or even cure esophageal mucosal injury.

Numerous studies reported that BILs were associated to esophageal inflammation in GERD patients, and distal BILs in RE patients were significantly lower than those in NERD patients.^[9–11,29,30]^ Overall, it is reasonable to hypothesize that BILs decrease in parallel with the severity of GERD, from healthy to NERD, and then RE. Consistent with above studies, our data revealed that patients with severe esophagitis had lower distal BILs than those with mild esophagitis and NERD, and that patients with severe esophagitis in acid reflux type had lowest distal BILs. However, our findings showed that there were no statistical significance in distal BILs between RE patients and NERD patients. Small sample size may be related with the results. Furthermore, long-term usage of PPIs might reduce difference of mucosal injury between 2 groups.

We found that distal BILs were negatively correlated to acid-related parameters, such as episodes of acid reflux, DeMeester score, and AET. These data, in keeping with previous findings,^[9,31,32]^ suggested a possible role of acid, although at physiological levels, in GERD. The mechanisms responsible for reflux perception are not yet fully understood. In our data, we also showed there was no correlation between BILs and episodes of nonacid reflux. In this regard, acid reflux remains more important determinant in the generation of symptoms, although MII-pH studies have shown that both acid and nonacid reflux can generate symptoms.^[23,24,33,34]^ We think BILs are related to the chemical clearance of the esophagus and concomitant with physiological levels of esophageal acid or nonacid exposure, but to our knowledge, we have no idea that how many episodes of nonacid reflux will be beyond the capacity of chemical clearance of esophagus. On the other hand, this finding maybe could explain partly why there was no response to PPI therapy in RGERD patients, possibly other factors such as visceral hypersensitivity or psychological disturbances may be more important than the severity of esophageal mucosal damage.

According to our results, we believe that a more in-depth pathophysiological evaluation of MII-pH tracings by adding of BILs could be of help to better investigate and identify patients with RGERD. However, there still were limitations of our study were: our study was retrospective, we could not collect enough information such as smoking, drinking, BMI, severity of reflux symptoms, and detailed information about other medications; all patients came from our single clinical center, the numbers were limited, and we lack of regular GERD patients and normal population.

In conclusion, based on our data, BILs were related with acid exposure and severity of esophagitis in RGERD patients, which was similar to that of regular GERD patients but may be more complex. Moreover, we believe that the assessment of esophageal BILs could represent a marker for acid or nonacid reflux induced changes to the esophageal mucosa. However, the results from our study warrant further research in RGERD patients to validate the measurement of BILs.
